# Challenge Accepted! a Critical Reflection on How to Perform a Health Survey Among University Students—An Example of the Healthy Campus Mainz Project

**DOI:** 10.3389/fpubh.2021.616437

**Published:** 2021-06-21

**Authors:** Jennifer L. Reichel, Thomas Rigotti, Ana Nanette Tibubos, Antonia M. Werner, Markus Schäfer, Dennis Edelmann, Daniel Pfirrmann, Nicole Deci, Manfred E. Beutel, Birgit Stark, Perikles Simon, Stephan Letzel, Pavel Dietz

**Affiliations:** ^1^Institute of Occupational, Social and Environmental Medicine, University Medical Center of the University of Mainz, Mainz, Germany; ^2^Department of Work, Organizational, and Business Psychology, Institute for Psychology, Johannes Gutenberg University Mainz, Mainz, Germany; ^3^Leibniz Institute for Resilience Research, Mainz, Germany; ^4^Department of Psychosomatic Medicine and Psychotherapy, University Medical Center of the Johannes Gutenberg University Mainz, Mainz, Germany; ^5^Department of Communication, Johannes Gutenberg University Mainz, Mainz, Germany; ^6^Department Sports Medicine, Rehabilitation and Disease Prevention, Institute of Sports Science, Johannes Gutenberg University, Mainz, Germany

**Keywords:** health survey, student health, health promoting university, health promotion, university students

## Abstract

**Background:** Universities represent an important setting of everyday life for health promotion. The *Healthy Campus Mainz* project aims to develop an evidence-based and comprehensive student health management program covering physical, mental, and social health promotion. Hence, an initial health survey was performed in order to identify the students' health concerns and resources. Up until now, it remains unclear which topics to choose in a health survey among university students and which strategies can be recommended to receive an acceptable response rate or representative student sample within a university setting. The present paper contributes to the call for the present research topic “Public Health Promotion in University Students” by describing methods for health assessment. Therefore, the current paper aims to give an empirical example on how to perform a health survey among university students, focusing on (1) choosing topics for the survey and (2) methodological considerations of how to reach the target population.

**Methods:** An online questionnaire including around 270 items was developed covering a comprehensive set of health topics. Participants were recruited via the university email. Mixed channels for survey promotion, such as lecture visits and social media, were used, accompanied by different monetary and non-monetary incentives. Descriptive analyses were performed to describe the sample.

**Results:** A total of 5,006 participants (out of 31,213 registered students) viewed the first page of the questionnaire; of whom, 4,714 continued further. After a manual data cleaning according to the predefined criteria, the final sample was 4,351, demonstrating a response rate of 13.9%. Students from different study disciplines participated. However, some study disciplines showed a low participation rate, hence, making the results not free from some bias.

**Discussion:** This survey is exceptional as it integrates a great variety of health aspects. The incentive strategy demonstrated promising results. Future research should try to improve target-group-specific recruitment strategies for the traditionally underrepresented groups, such as males and specific study disciplines. This would not only include advancing marketing strategies, but also refining the incentive strategy.

## Introduction

According to the World Health Organization, health is not just a state but, rather, “a resource for everyday life” (1). It is created and lived by people within the settings of their everyday life, where they learn, work, play, and love (1), underlining the interconnectedness between individuals and their environments. In the *Okanagan Charter* of 2015, an international expert group emphasized that universities are important settings of people's everyday life for health promotion (2). They further claimed that the collective population of university students would be particularly relevant from a public health point of view. They argued that health promotion in university students would not only be favorable for the health of the target population, but since university students are the decision-makers, leaders, and also parents of tomorrow, health promotion may also benefit the society as a whole (2). The high societal relevance of health promotion for different settings can also be seen in the recent developments in the legislations. In 2015, the German Government passed the so called “*prevention law*,” aiming to support health promotion and prevention in different settings of everyday life (3). Accordingly, the statutory health insurances have to spend a set amount of money (around 7€) for each insurant for health promotion and prevention projects in different settings of everyday life.

Supported with financial resources of a statutory health insurance in the framework of the prevention law, the *Healthy Campus Mainz* project was initiated in 2018. It is an interdisciplinary research project, aiming to create, implement, and evaluate an evidence-based, sustainable, and holistic student health management program for ~32,000 students at the University of Mainz. This interdisciplinary approach is essential in order to cover a great variety of aspects about student health. The project, therefore, includes experts from the following disciplines: occupational, social, and environmental medicine; work, organizational, and business psychology; psychosomatic medicine and psychotherapy; media science; and sports medicine.

A crucial part of developing an evidence-based health management program tailored to the needs of the local students is to perform a health survey among students in order to specify areas of interest and identify potential risk groups for poor health within the target population. The identification of risk groups can be based on study-related aspects such as field of study, time studying at a university (4), or individual differences in psychosocial personal factors such as personality, behavioral habits, or socioeconomic status. Such information may enable the stakeholders of student health management programs to understand the distinctive needs of its own students, tailor health promotion interventions, and develop policies accordingly. Also, Baik et al. (5) already pointed out how important it is to involve students in the design and implementation of such health programs as this underscores that they are the “experts” of their own needs. Looking at the reports on surveys addressing the university students' health, Kunttu et al. (6) conducted a students' health survey for Finland, Holt and Powell (4) in the UK, and Wörfel et al. (7) in Germany. Kunttu et al. (6) performed an online national survey among Finnish university students (*n* = 1,829) with 126 questions including a broad set of topics regarding physical, mental, and social health. The purpose of their survey was to map the university students' wellbeing, study ability, and health issues. The 60-item online survey by Holt and Powell was developed to examine the health behaviors and health needs of students (*n* = 3,683) at a UK urban University with a focus on seven key topic areas (4). These topic areas were chosen based on national and local priorities. Under the umbrella of the *University Health Report* project in Germany, a health survey was developed that can be used and adapted by any other university aiming for health assessment of their students (8). The latest published report, for example, performed at two German universities (*n* = 1,707) assessed the “strains, resources, health indicators, health behavior, and health risks” as part of a periodical health monitoring (7).

The variety of covered topics in the previous studies, however, is still expandable. Moreover, there is no sufficient consensus on what topics should be included. Seemingly, it also remains unclear which strategies can be recommended to receive an acceptable response rate or representative student sample within a university setting. The present paper contributes to the call for the present research topic, “Public Health Promotion in University Students,” by describing methods for health assessment. Therefore, the current paper seeks to answer the following questions:

1. Survey content: Which relevant topics should be included in a health survey among university students?
2. Methodological Considerations: How can the target group of university students be reached in order to gain a large and representative sample?
  2.1 Survey method
  2.2 Questionnaire design
  2.3 Recruitment and survey promotion
  2.4 Incentive strategy

As many universities face similar challenges, the present paper aims to address the above-mentioned questions and to provide an example on how to perform an effective health survey among university students. These suggestions are based on our experience, and are meant to provide a platform for discussions and suggestions for future studies.

## Materials and Methods

### Survey Content

The core of planning a health survey is to decide on the topics that should be included. The content of a student health survey should represent and address the specific goals of the overall project and university, respectively. These may vary from university to university and from project to project. Therefore, prior to planning a health survey, the stakeholders need to clarify the specific goals of their undertaking. Within *the Healthy Campus Mainz* project, we pursued a comprehensive approach aiming to address a wide range of health-related topics. We followed the World Health Organization's understanding of health as consisting of the physical, mental, and social dimensions of wellbeing (9), and can name mental health, physical activity, and media use as some specific examples of our targeted health topics. Additionally, in accordance with the prevention law, we aimed to identify the potential health-related risk groups within the student population concerning their sociodemographic factors, field of study, and specific/vulnerable phases during enrollment at the University. Furthermore, as stated in several articles, determinants of health (behavior), such as personality factors and structural conditions, are relevant aspects to be investigated (7, 10, 11). This is also important as understanding why individuals tend to engage in a specific health behavior contributes to an evidence-based planning of health-promoting interventions. Only by addressing the relevant determinants that have a causal relationship with health conditions can interventions be effective (12). Based from reviewing the literature and former health surveys (4, 8), the interdisciplinary research team of *Healthy Campus Mainz* decided to employ established and validated instruments wherever feasible, and to minimize the use of self-developed scales. The good psychometric quality of assessment tools enhances the reliability and validity of the gathered information. In addition, it makes the findings comparable to other studies and allows generalization. It was particularly the aim to cover new aspects that had not been represented sufficiently in other surveys, namely, media use and utilization of medical prevention services. We also wanted to examine the determinants of health, and to, thereby, portray a broad view on health. A recent systematic umbrella review revealed that these topics often have been neglected in former studies among university students (13). Consequently, the following topics were covered in the survey consisting of ~270 items (a detailed list of included scales can be found in the Supplementary Table 1):

- health condition
  ◦ overall health, mental health, chronic diseases, and disability
- health behavior
  ◦ physical activity, presenteeism/absenteeism, diet, media use, procrastination, substance use (including neuroenhancement), vaccination, and oral hygiene
- determinants of health condition and behavior
  ◦ determinants related to: sociodemographic factors, biography, social factors, individual psychological factors, health literacy, and structural conditions (resources and demands)

### Methodological Considerations

Many different aspects play together when it comes to reaching the target group while aiming for a large and representative sample. In the following sections, we will describe our strategy accordingly.

#### Survey Method

Choosing the appropriate survey method requires careful consideration. The *Healthy Campus Mainz* team decided to use an online questionnaire since the target group seemed to be familiar with online surveys. Students spend a great amount of their time online for private and study-related purposes (14). Online surveys provide a great opportunity to reach many people in a university setting (15). In addition, the monetary savings of an online survey were a reason for this format, as there is no need to print the questionnaires on paper (16) and costs for typing in data are circumvented.

To reduce concerns about the anonymity and adherence to the privacy policy of an online survey that may prevent people from participating, it was stressed in the introduction of the survey that it adheres to the privacy policy and that it is strictly anonymous.

#### Questionnaire Design

Choosing an adequate length of a survey generally depends on the content and context in which the survey is performed. The estimated time for completion of the ~270-item-survey was 35–45 min. This still seemed to be an adequate amount of time students would be willing to provide if the survey is connected to an appealing incentive strategy. Even though one might argue that a shorter survey would increase the likelihood for a higher response rate (17), to us, the decision had to be prioritized according to the content. As previously mentioned, our focus was to include many different facets of health and link them to a variety of potentially interacting determinants (12). In order to minimize bias in the results, it is important to design the questionnaire carefully (18). Therefore, the construction of the questionnaire needs to take the further described aspects into account. Validated short forms of established standardized questionnaires, if applicable, should be used preferably instead of long, time-consuming versions. This brings the advantage of the quick assessment of a variety of target variables and, at the same time, makes the overall questionnaire shorter, hence, reducing preparation time and making dropout less likely. Furthermore, using established standardized questionnaires goes along with higher objectivity and reliability. If available, the normative data of these questionnaires also allow comparison with other populations and enable the generalization of the findings in terms of validity aspects (19).

The inclusion of a moderation text throughout the questionnaire that provides easy transitions from question to question was a crucial part of the questionnaire design. To keep participants motivated, a process bar at the bottom of the page was included. In addition, motivating phrases and Graphic Interchange Formats (GIFs) were incorporated. The questionnaire was designed using the software *Unipark*. Prior to performing the survey, 12 students participated in a pre-test, which resulted in minor adaptations of the questionnaire according to their suggested feedback.

#### Recruitment Strategy and Survey Promotion

The timing of the survey also requires consideration in order to receive many survey responses. We chose to invite participants in the middle of the semester until the end, because the workload would seem more representative of their typical study demands and its relation to health (behavior) as opposed to the beginning of the semester. This might vary, though, between different university systems in different countries. The survey was open for participation until the beginning of the semester break when students typically still take exams or work on assignments.

Conducting a health survey among university students aiming for a high response rate and representativity of the whole population was, as already mentioned, important to us. That is why a carefully considered recruitment strategy was necessary. Overall, the recruitment took place for 7 weeks. In order to reach as many students as possible, an email including a link to the survey was sent to the whole student body via the system mail of the University of Mainz. Thereby, all students who were currently enrolled at the University received an email to their account where they would normally receive important notifications (e.g., about their grades). The emails were tailored to the target audience and the incentives were emphasized in order to increase motivation. In addition, the emails highlighted the overall project goals of *Healthy Campus Mainz*, the need for student participation and the health benefits for students regarding long-term study environment changes at the university. Consequently, four reminder emails were sent when the participation rate seemed to drop or almost stagnated.

As part of our survey promotion strategy, we additionally tried to serve many different communication channels to remind the students of our survey and to motivate them for participation. That is why, secondly, large lectures were visited by different members of the *Healthy Campus Mainz* team. Students were invited to participate in the survey according to a protocol by introducing the overall project goals and highlighting the incentives. Similarly, lecturers of smaller classes were asked to show an invitation during their lecture on the classroom screen. Thirdly, survey promotion on campus took place by placing marketing material like posters, leaflets, post cards, and stickers on pinboards and common areas, such as the cafeteria. In the process of designing the marketing material, a great attention had to be paid to creating an appealing design, catchy slogans, and to provide precise and the most relevant information. Face-to-face promotion on campus took place, and a chillout-area on campus was installed where students were invited to fill in the questionnaire on tablets or their smartphones in private. Handing out fresh fruits served as an incentive. Furthermore, press articles in the local news were released that announced the health survey.

Another important part of the promotional strategy was the use of social media, as it has been shown to be an appropriate strategy for reaching a young and large sample (20). A project Instagram account was launched shortly before the start of the survey. In order to gain followers, the marketing material for the survey included the name of the project's Instagram page, and during the recruitment in the lecture, people were invited to follow the page. On Instagram, regular posts and so called “stories” were posted to invite participants and give updates about the current participation rate. Besides this, other stakeholders on campus shared the information on the survey on their social media channels (Instagram and Facebook) as part of the survey promotional strategy.

#### Incentive Strategy

In order to maximize participation, it was crucial to have an incentive strategy which appeals to the broad masses of the student population. That is why a small survey among students (*n* = 24) about the incentive strategy was conducted in advance to identify what would motivate them for participation. It turned out that they would prefer a mix of small monetary and non-monetary incentives. Zheng et al. (21) also found that a mix of incentives would be an important factor in the recruitment of participants for health surveys. Consequently, to reach a wide spectrum of different people, a mixed incentive strategy was chosen. The main incentive was the following: *If 5,000 people complete the questionnaire, 1,000*€ *will be donated to the child cancer aid of Mainz*. This charity organization was chosen since it is directly linked to the topic of health and, desirably, it could create an emotional response. In addition, the promise of a charitable donation can activate the respondents' altruism (22). Throughout the whole survey conduct, the students were informed via reminder emails and social media about the current number of completed surveys in order to further promote participation. Besides this, lottery of gift cards for local gastronomy providers and an online store functioned as monetary incentives. We included 13 gift cards for local gastronomy providers (7 × 24€ and 6 × 40€). In addition, we offered 15 gift cards for an online store (5 × 100€, 5 × 50€, and 5 × 20€).

## Results

Of the around 32,000 students at the University of Mainz, 5,006 participants viewed the first page of the online-questionnaire; of whom, 4,714 continued further with the survey. Answering the first question of the questionnaire regarding health was a prerequisite in order to be included for further analyses. Participants who completed the questionnaire in <20 min were excluded since this appeared not to be enough time in order to fill in the questionnaire conscientiously/carefully. Also, cases with values that were not in the value range were controlled manually and excluded if they did not seem plausible. After manual data cleaning according to the just described predefined criteria, the final cleaned sample was 4,351, demonstrating a response rate of 13.9% of the total student population at the University of Mainz. Eventually, 3,914 participants fully completed the survey. On average, participants spent 43.5 min to complete the survey.

**Figure 1:**
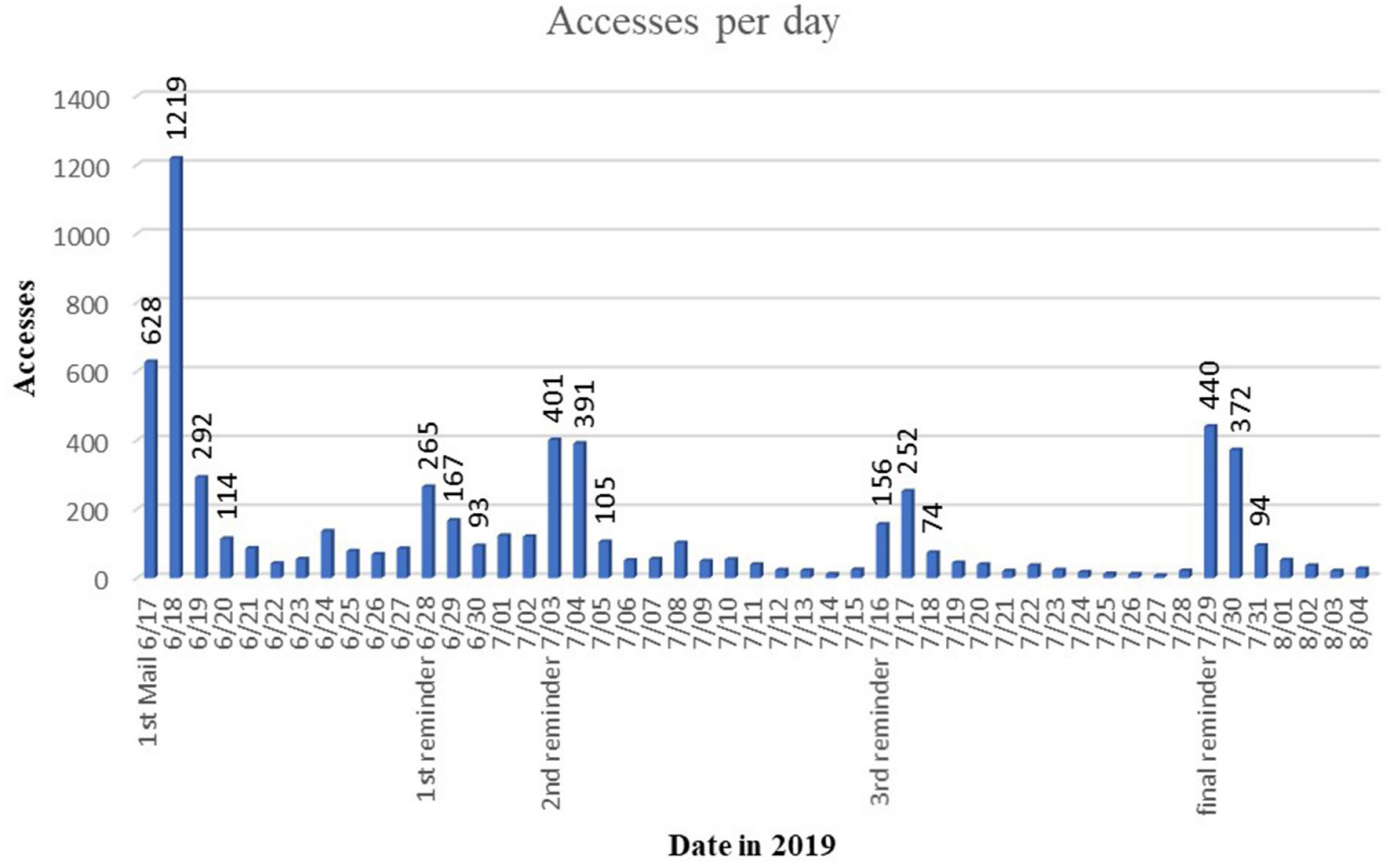
Accesses to the online survey per day during the whole survey period.

The time of respondence seemed strongly dependent on the reminder emails that were sent. Accordingly, the first invitation mail resulted in the most completed questionnaires. Figure 1 gives an overview of the response/access over time and dates when reminders were distributed. Note, that the first reminder email did not result in as many responses as typically would have been expected due to a technical error in the distributed email that could have disturbed the students. The final reminder was sent at the beginning of the semester break, when students do not have any classes but some have tests and assignments. This resulted in more participants than the previous reminders.

The majority (*n* = 3,065; 70.5%) of the participants were female, 28.6% (*n* = 1,246) were male, and 0.9% (*n* = 39) identified themselves as diverse. Compared to the gender distribution of the University of Mainz as a whole, women were overrepresented by 11.5% percentage points. The mean age was 23.8 and, thereby, approximately representative of the whole student body of the University of Mainz that has a mean age of 24.7. Table 1 provides an overview of the participant characteristics.

**Table 1:** Participant characteristics.

**All, *n* (response rate in %)**	4,351 (13.9)
**Gender**, ***n*** **(%)**	
Female	3,065 (70.5)
Male	1,246 (28.6)
Diverse	39 (0.9)
**Age, years (MW ± SA)**	16–73 (23.8 ± 4.4)
**University semesters, (MW ± SA)**	1–45 (7.1 ± 4.9)
**Degree**, ***n*** **(%)**	
Bachelor's degree	2,261 (52.0)
Master's degree	920 (21.1)
State examination	977 (22.5)
PhD	146 (3.4)

**Figure 2:**
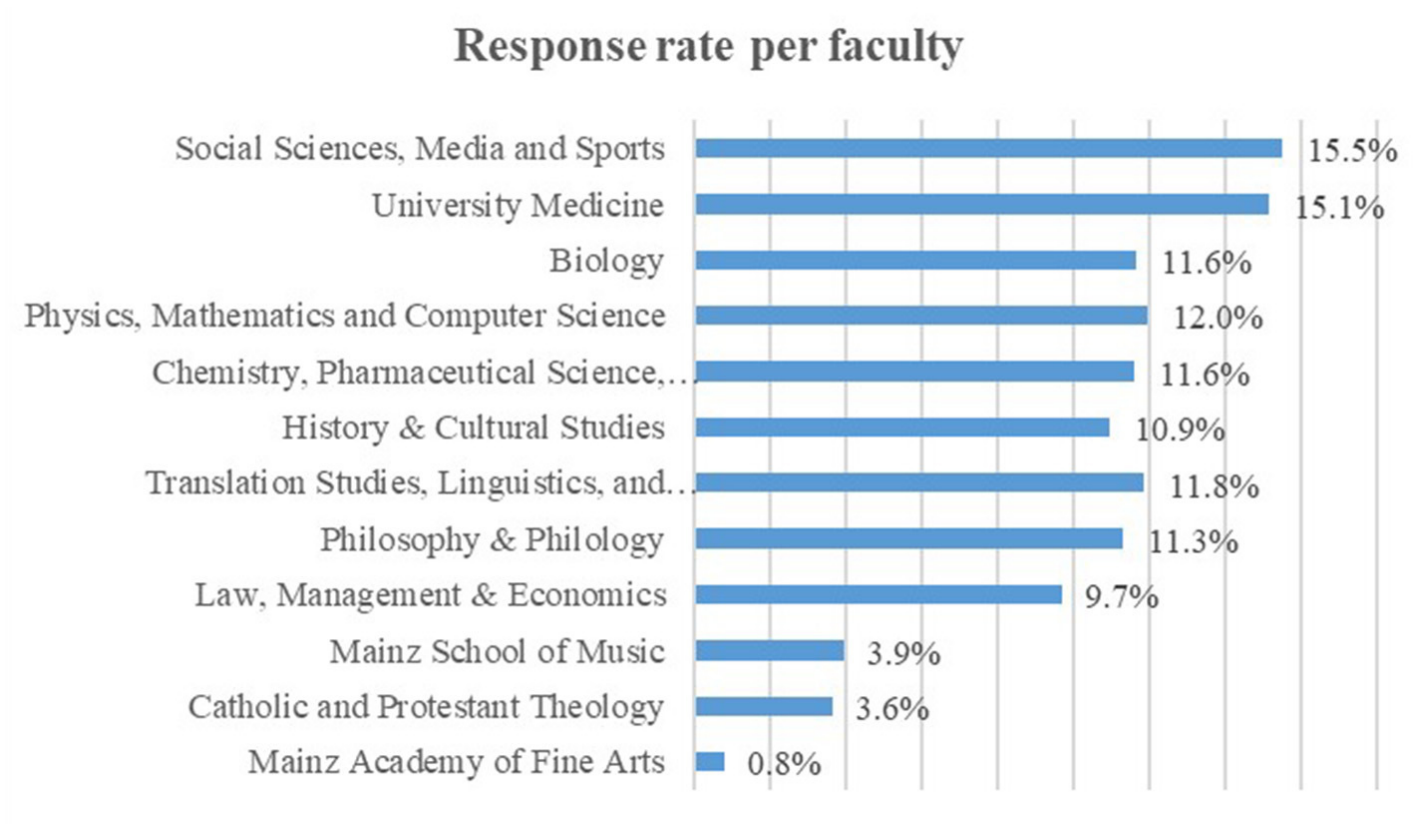
Survey response rate distributed for faculty.

Participants were enrolled in different degree levels. 52.0% (*n* = 2,261) pursued a Bachelor's degree, 21.1% (*n* = 920) a Master's degree, 22.5% (*n* = 977) a state examination, and 3.4% (*n* = 146) a PhD. Other degree levels that will soon expire or are not very common in the German system were only represented with a very few students. Students from all faculties and from many different study disciplines, such as social sciences (e.g., psychology), economics, and law, participated. A large number of students from the sample was from the faculties of *Philosophy and Philology* (*n* = 601; 13.8%) and *University Medicine* (*n* = 582; 13.4%). Figure 2 shows the response rates of each faculty in relation to all students of the according faculty at the university. Despite the fact that we reached all faculties, it shows that we did not reach students in every single faculty to the same extent. The faculty *Social Sciences, Media, and Sports* had the highest response rate with 15.5%, followed by the faculty of *University Medicine* with 15.1%. The *Mainz Academy of Fine Arts* (0.8%), the *Mainz School of Music* (3.9%), and the *Faculty of Catholic and Protestant Theology* (3.6%) showed the lowest response rates.

## Discussion

The aim of the current paper was to provide an example of how to perform a health survey among university students. This article should not be seen as a guideline that is “set into stone” but rather as an aid with useful hints for other universities in their similar undertakings.

The health survey at the University of Mainz had a slightly higher response rate (13.9%) compared to a similar health survey by Holt and Powell (4) that resulted in a response rate of 10%. Similar health topics were assessed but they additionally asked for health care utilization, which could be interesting for further investigations at the University of Mainz as well. In Germany, the response rate of a previous health survey was 9% (23). Compared to other studies, the sample of this health survey is quite large, despite the length of the survey. A possible explanation for this could have been the promotion of the survey via multiple channels and the differentiated incentive strategy. At the Technical University Kaiserslautern (24), 1,383 participated; and at Freie Universität Berlin, 2,620 participated (25). One has to note that some other studies do not report completion or response rates, which makes comparison more difficult. Despite the fact that response rates seem to be an important aspect, the pursuit of a large sample is also important when one aims to perform complex statistical analyses. This is most certainly the case in our project as we do not only want to assess the health status and behavior, but beyond that, identify potential determinants of health status as well as behavior. This goes along with needing more variables that are assessed in the questionnaire. Therefore, we had to compromise the risk of a lower response rate due to the survey length with a more comprehensive data. We also aspire longitudinal study-designs in the future. In the meantime, one publication about health information seeking behavior of university students that is based on the data collected through this described survey has already been published by Schäfer et al. (26). It will be followed by several other studies with different focus areas, such as mental health (27, 28).

When interpreting the results, it needs to be considered that the time frame during which the data were collected, the students might have been exposed to a different work load compared to other phases of the semester due to exams and assignments. If other universities consider to transfer parts of the approach to their university, they need to take their overall project goals, specific characteristics of their university, and also, the available resources into account and adapt the approach accordingly.

### Potential Pitfalls and Further Considerations

The survey was only conducted in German, hence, international students, who are not fluent in German, were not able to take part in the survey. Therefore, it is possible that problems of this specific group, which may relate to their different cultural background (29), could not have been detected. Then again, the administration of multi-lingual questionnaires requires measurement equivalence between the applied language versions and the different participating cultural groups which is not sufficiently tested for in most cases (30). Potentially limiting the results is the self-reporting assessment of the survey. Especially in regard to sensitive health topics (e.g., illicit drug use), socially desirable answers are possible (31). It has been found that sensitive questions are not always answered correctly since people tend to give socially desired responses (31).

Since a large number of the participants were female and from certain fields of studies, the findings of this survey might be more valid for these groups and not generalizable. Especially in the field of mental health, sex-difference are common phenomena affecting reporting and help-seeking behavior (32). The higher response rate of females, however, seems to be a common issue in online studies among students (33) and in health surveys (34). Having this in mind, researchers who plan to do similar health surveys could intensify their efforts to recruit male participants and the typically underrepresented fields of studies by investing more in survey promotion. In our particular case, for instance, the Faculty of Fine Arts is not located on the same campus as the other ones. This could be an explanation for why other faculties on campus could be better reached by the use of face-to-face recruitment and lecture visits. One also has to note, as a limitation of our survey the data, that statistical analyses to study group differences cannot be performed with certain field of studies when the sample size is too little.

Future health surveys should try to improve target group specific recruitment and incentive strategies for traditionally underrepresented groups such as males and students from certain study disciplines even further. This would not only include advancing marketing strategies, but also refining the incentive strategy, for example, by including sub-goals within the donation goal. Another interesting option to study different incentives would be to see how many students participated with no incentive (email one), with the charity incentive (email two), and with the gift cards (emails three and four). However, the feasibility of this strategy would need to be taken into account based on the rest of the marketing strategy. Besides this, specific attention should be paid to the ways contacted persons converted to participants by tracking or assessing the source that made people actually participate.

## Conclusions

Aspects that should be taken into consideration throughout the planning process of a health survey among university students are the following:

- Planning the topics of the survey in accordance with your project goals and based on the circumstances at your University.
- Using short, valid forms of established questionnaires, if applicable.
- Using an online survey is appropriate for the target group of students. Email invitations to the whole target population seem useful for recruitment.
- For survey promotion using many different communication channels, in particular lecture visits, social media, and face-to-face on campus promotion. Focus promotion efforts on typically underrepresented groups.
- Offering a variety of incentives and making them interesting or emotionally relatable to the target group.

To conclude, with this article, we wanted to share some of our “lessons-learned” from the *Healthy Campus Mainz* project and provide a platform for discussion. We hope that our suggestions are helpful for those planning health surveys among students, and that others share their experience and best-practice cases to guide an evidence-based process. We invite other researches in the field to also report their strategies for survey development and promotion that seemed beneficial but, also, explicitly the ones that did not work out and innovative ideas are needed instead.

## Data Availability Statement

The raw data supporting the conclusions of this article will be made available by the authors, without undue reservation.

## Ethics Statement

Ethical clearance was received by the Ethical Committee of the Medical Association (Ärztekammer) Rhineland-Palatinate (No. 2019-14336). Written informed consent from the participants' legal guardian/next of kin was not required to participate in this study in accordance with the national legislation and the institutional requirements.

## Author Contributions

JR, TR, and PD analyzed the data. JR and PD were the major contributors in the writing process. All authors were involved in the development and performance of the survey. All authors contributed in writing the manuscript. All authors read and approved the final manuscript.

## Conflict of Interest

The authors declare that the research was conducted in the absence of any commercial or financial relationships that could be construed as a potential conflict of interest.
