# Supplementary material for: Challenge Accepted! a Critical Reflection on How to Perform a Health Survey Among University Students—An Example of the Healthy Campus Mainz Project
Source: Front Public Health. 2021 Jun 21;9:616437. doi: 10.3389/fpubh.2021.616437 (PMC8255799; doi:10.3389/fpubh.2021.616437)
Supplement: Supplementary file 1 [file Table_1.pdf]

Supplemental Material **Healthy Campus Mainz – Questionnaire Content**

**Dimension**	**Variable**	**Scale/ Reference**	**Items**
**Health condition**	General Health	Nübling, 2005 (1)	1
	Chronic Disease (mental)	Self-developed	1
	Chronic Disease (physical)	Self-developed	1
	Disability	Self-developed	1
	Anthropometry	Self-developed	2
	Depressive Symptoms	PHQ-9; Kroenke et al., 2001 (2)	9
	General Anxiety	GAD-2; Döring & Bortz, 1993 (3)	2
	Somatic Symptoms	SSS-8; Gierk et al., 2014 (4)	8
	Social Anxiety	Mini-SPIN; Wiltink et al., 2017 (5)	3
	Eating Disorder	EDE-Q8; Kliem et al., 2016 (6)	3
	Emotional Exhaustion	MBI-SS; Gumz et al., 2013 (7)	5
**Health Behavior**	Presenteeism	Töpritz et al. 2016 (8)	1
	Absenteeism	Töpritz et al. 2016 (8)	1
	Physical Activity & Sedentary Behavior	IPAQ short version; Craig et al., 2003 (9, 10)	7
	Convenience Behavior	CBQ; Dreher et al., 2019 (11)	15
	Healthy Diet	Self-developed	1
	Fruit and Vegetable Consumption	European Comission, 2013 (12); Fehr et al., 2017 (13)	4
	Media Use (General Use, (mobile) internet use, used content)	Adapted from Stark et al., 2017 (14)	6
	Health-related information sources (On- und Offline) & Media Use	Adapted from Baumann & Czerwinski, 2015 (15); Marstedt, 2018 (16)	8
	Health Literacy	Schaeffer et al., 2017 (17) (shortened version)	4
	Health-Related Opinion Leadership	Childers, 1986 (18); Rössler, 2011 (19)	1
	Internet Addiction	AICA-short; Wölfling et al., 2012 (20)	6
	**Substance Use**		
	Alcohol Use	AUDIT; Bush et al., 1998 (21)	3
	Smoking	University Health Report; Töpritz et al. 2016 (8)	1
	Illegal Drugs	Self-developed	1
	Pharmacological Neuroenhancement	Dietz et al., 2016 (22)	2
	Procrastination	GPS-K; Klingsieck & Fries, 2012 (23)	9
	Vaccination	Self-developed	1

**Dimension**	**Variable**	**Scale/ Reference**	**Items**
	Teeth hygiene	Self-developed	1
**Determinants**	**Biography**		
	adverse childhood experience	ACE; Wingenfeld et al., 2011 (24)	10
	Mobbing (currently)	MOB-K; adapted from Pfaff et al., 2007 (25)	4
	(Cyber) Mobbing at school	SOEP; DIW (26)	1
	(Cyber) Mobbing (aktively)	SOEP; DIW (26)	2
	Sexual Harassment	Self-developed	1
	**Social Factors**		
	Loneliness	UCLA; Döring & Bortz, 1993 (3)	3
	Social Support	SALSA; Rimann & Udris, 1997 (27)	3
	**Individual Psychological Factors**		
	Gender Identity	PAQ-8; Runge et al., 1981 (28)	8
	Self-Acceptance	Ryff, 1989; (29) Ryff & Keyes, 1995 (30)	3
	Self-Criticism	DEQ-SC4; Rudich et al., 2008; (31) Tibubos et al., 2020 (under review)	4
	Sensation seeking	SOEP; DIW (26)	1
	Risk Attitude – health related	DOSPERT; Adapted from Johnson et al., 2004; (32) Blais et al., 2006 (33)	6
	Cognitive Irritation	Mohr et al., 2005 (34)	
	**Personal Resources**		
	Planning competence and competence for self-motivation	Dettmers & Clauß, 2018 (35)	8
	Self-Efficacy	Self-Efficacy Scale; Adapted from Rigotti et al., 2008 (36)	6
	Emotion Regulation	ERQ; Abler & Kessler, 2011 (37)	4
	**Structural Ressources**		
	Autonomy	BARI-S; Gusy & Lohmann, 2014 (38)	6
	**Structural study-related Demands**		
	Quantitative Demands	COPSOQ; Nübling, 2005 (1)	1
	Performance Pressure	Fend, 1997 (39)	5
	Qualitative Excessive Demands	BARI-S; Gusy et al., 2016 (40)	2
	Competition	Fend, 1997 (39)	4
	Perceived Employability	Self-developed	1

**Dimension**	**Variable**	**Scale/ Reference**	**Items**
	**Coping behavior**		
	Self-endangering behavior/coping	Krause et al., 2015 (41)	4
	Coping via use of structural resources	In style of Daniels & Harris, 2005 (42)	4
**Sociodemographics**	Gender	Internal University Item	1
	Age	Internal University Item	1
	Field of study	Internal University Item	1
	Degree	Internal University Item	1
	Semester	Internal University Item	1
	Relationship Status	Internal University Item	1
	Marital Status	Internal University Item	1
	Children	Internal University Item	1
	Parents‘ Educational Background	Internal University Item	1
	Nationality	Internal University Item	1
	Migrant Background	Internal University Item	1
	Personal Migration Experience	Internal University Item	1
	Housing Situation	Internal University Item	2
	Distance to (parental) home	Internal University Item	1
	Financial Situation	Internal University Item	1

References

1. Nübling M. *Methoden zur Erfassung psychischer Belastungen: Erprobung eines Messinstrumentes (COPSOQ) ; [Abschlussbericht zum Projekt "Methoden zur Erfassung psychischer Belastungen - Erprobung eines Messinstrumentes (COPSOQ)" - Projekt F 1885*. Bremerhaven: Wirtschaftsverl. NW Verl. für Neue Wiss (2005). 141 p.
2. Kroenke K, Spitzer RL, Williams JB. The PHQ-9: validity of a brief depression severity measure. *J Gen Intern Med* (2001) **16**:606–13. doi:10.1046/j.1525-1497.2001.016009606.x
3. Döring, N., & Bortz, J. Psychometrische Einsamkeitsforschung: Deutsche Neukonstruktion der UCLA Loneliness Scale [Psychometric research on loneliness: A new German version of the University of California at Los Angeles (UCLA) Loneliness Scale]. *Diagnostica* (1993) **39**:224–39.
4. Gierk B, Kohlmann S, Kroenke K, Spangenberg L, Zenger M, Brähler E, et al. The somatic symptom scale-8 (SSS-8): a brief measure of somatic symptom burden. *JAMA Intern Med* (2014) **174**:399–407. doi:10.1001/jamainternmed.2013.12179
5. Wiltink J, Kliem S, Michal M, Subic-Wrana C, Reiner I, Beutel ME, et al. Mini - social phobia inventory (mini-SPIN): psychometric properties and population based norms of the German version. *BMC Psychiatry* (2017) **17**:377. doi:10.1186/s12888-017-1545-2
6. Kliem S, Mößle T, Zenger M, Strauß B, Brähler E, Hilbert A. The eating disorder examination-questionnaire 8: A brief measure of eating disorder psychopathology (EDE-Q8). *Int J Eat Disord* (2016) **49**:613–6. doi:10.1002/eat.22487
7. Gumz A, Erices R, Brähler E, Zenger M. Faktorstruktur und Gütekriterien der deutschen Übersetzung des Maslach-Burnout-Inventars für Studierende von Schaufeli et al. (MBI-SS). *Psychother Psychosom Med Psychol* (2013) **63**:77–84. doi:10.1055/s-0032-1323695
8. Töpritz, K., Lohmann, K., Gusy, B., Farnir, E., Gräfe, C. & Sprenger, M. *Wie gesund sind Studierende der Technischen Universität Kaiserslautern? Ergebnisse der Befragung 06/15*. Berlin: Freie Universität Berlin.: Schriftenreihe des AB Public Health: Prävention und psychosoziale Gesundheitsforschung: Nr. 01/P16 (2016). 163 p.
9. Craig CL, Marshall AL, Sjöström M, Bauman AE, Booth ML, Ainsworth BE, et al. International physical activity questionnaire: 12-country reliability and validity. *Med Sci Sports Exerc* (2003) **35**:1381–95. doi:10.1249/01.MSS.0000078924.61453.FB
10. International Physical Activity Questionnaire. *International Physical Activity Questionnaire. Short last 7 days self-administered format - german version* (2016). Available from: https://docs.google.com/viewer?a=v&pid=sites&srcid=ZGVmYXVsdGRvbWFpbnx0aGVpcGFxfGd4OjNiMGRjYjU3NzE5Nzc0ZGY Access at 08th of May 2020
11. Dreher M, Hoffmann SW, Brendel C, Heser D, Simon P. Convenience Behavior and Being Overweight in Adults: Development and Validation of the Convenience Behavior Questionnaire. *Front Public Health* (2019) **7**:20. doi:10.3389/fpubh.2019.00020

12. European Comission. *European Health Interview Survey (EHIS wave 2): Methodological manual 2013 edition*. Luxembourg: Publications Office of the European Union (2013). 111 p.
13. Fehr A, Lange C, Fuchs J, Neuhauser, H, Schmitz, R. Gesundheitsmonitoring und Gesundheitsindikatoren in Europa. *Journal of Health Monitoring* (2017) **2**. doi:10.17886/RKI-GBE-2017-004.2
14. Stark B, Magin M, Jürgens P. *Ganz meine Meinung? Informationsintermediäre und Meinungsbildung - eine Mehrmethodenstudie am Beispiel von Facebook*. Düsseldorf: Landesanstalt für Medien Nordrhein-Westfalen (LfM) (2017). 259 p.
15. Baumann, E., & Czerwinski, F. "Erst mal Doktor Google fragen? Nutzung neuer Medien zur Information und zum Austausch über Gesundheitsthemen,". In: Böcken J, Braun B, Meierjürgen R, editors. *Gesundheitsmonitor 2015: Bürgerorientierung im Gesundheitswesen ; Kooperationsprojekt der Bertelsmann Stiftung und der BARMER GEK*. s.l.: Verlag Bertelsmann Stiftung (2015). 57-79.
16. Marstedt G. *Das Internet: Auch Ihr Ratgeber für Gesundheitsfragen. Bevölkerungsumfrage zur Suche von Gesundheitsinformationen im Internet und zur Reaktion der Ärzte.* Gütersloh: Bertelsmann Stiftung. (2018).
17. Schaeffer D, Berens E-M, Vogt D. Health Literacy in the German Population. *Dtsch Arztebl Int* (2017) **114**:53–60. doi:10.3238/arztebl.2017.0053
18. Childers TL. Assessment of the Psychometric Properties of an Opinion Leadership Scale. *Journal of Marketing Research* (1986) **23**:184–8. doi:10.1177/002224378602300211
19. Rössler P. *Skalenhandbuch Kommunikationswissenschaft*. Wiesbaden: VS Verlag für Sozialwissenschaften (2011).
20. Wölfling K. Construction of a Standardized Clinical Interview to Assess Internet addiction: First Findings Regarding the Usefulness of AICA-C. *J Addict Res Ther* (2012). doi:10.4172/2155-6105.S6-003
21. Bush. The AUDIT Alcohol Consumption Questions (AUDIT-C): An Effective Brief Screening Test for Problem Drinking (1998).
22. Dietz P, Soyka M, Franke AG. Pharmacological Neuroenhancement in the Field of Economics-Poll Results from an Online Survey. *Front Psychol* (2016) **7**:520. doi:10.3389/fpsyg.2016.00520
23. Klingsieck KB, Fries S. Allgemeine Prokrastination. *Diagnostica* (2012) **58**:182–93. doi:10.1026/0012-1924/a000060
24. Wingenfeld K, Schäfer I, Terfehr K, Grabski H, Driessen M, Grabe H, et al. Reliable, valide und ökonomische Erfassung früher Traumatisierung: Erste psychometrische Charakterisierung der deutschen Version des Adverse Childhood Experiences Questionnaire (ACE). *Psychother Psychosom Med Psychol* (2011) **61**:e10-4. doi:10.1055/s-0030-1263161
25. Pfaff, H., Bentz, J. & Brähler, E. Die Skala „Mobbingintensität der Kolleginnen und Kollegen" (MOB-K): Teststatistische Überprüfung an einer repräsentativen Bevölkerungsstichprobe. *Psychosozial* (2007) **30**.

26. Deutsches Institut für Wirtschaftsforschung e.V. *Sozio-oekonomisches Panel* (2020) [cited 2020 May 29]. Available from: https://www.diw.de/de/diw_01.c.615551.de/forschungsbasierte_infrastruktureinrichtung__sozio-oekonomisches_panel__soep.html
27. Rimann, M., & Udris, I. "Subjektive Arbeitsanalyse: Der Fragebogen SALSA,". In: O. Strohm, & E. Ulich, editor. *Unternehmen arbeitspsychologisch bewerten. Ein Mehr-Ebenen-Ansatz unter besonderer Berücksichtigung von Mensch, Technik und Organisation*. Zürich, Switzerland: vdf Hochschulverlag (1997). p. 281–98.
28. Runge, T. E., Frey, D., Gollwitzer, P. M., Helmreich, R. L. & Spence, J. T. Masculine (instrumental) and feminine (expressive) traits: A comparison between students in the United States and West Germany. *Journal of Cross-Cultural Psychology* (1981) **12**:142–62.
29. Ryff CD. Happiness is everything, or is it? Explorations on the meaning of psychological well-being. *Journal of Personality and Social Psychology* (1989) **57**:1069.
30. Ryff, C. D., & Keyes, C. L. The structure of psychological well-being revisited (1995) **69**:719–27.
31. Rudich Z, Lerman SF, Gurevich B, Weksler N, Shahar G. Patients' self-criticism is a stronger predictor of physician's evaluation of prognosis than pain diagnosis or severity in chronic pain patients. *J Pain* (2008) **9**:210–6. doi:10.1016/j.jpain.2007.10.013
32. Johnson, J. G., Wilke, A., & Weber, E. U. A domain-specific scale measuring risk perceptions, expected benefits, and perceived-risk attitude in German-speaking populations. *Polish Psychological Bulletin*.
33. Blais, A-R. and E. U. Weber. A Domain-specific Risk-taking (DOSPERT) Scale for Adult Populations. *Judgment and Decision Making* (2006) **1**:33–47.
34. Mohr G, Rigotti T, Müller A. Irritation - ein Instrument zur Erfassung psychischer Beanspruchung im Arbeitskontext. Skalen- und Itemparameter aus 15 Studien. *Zeitschrift für Arbeits- und Organisationspsychologie A&O* (2005) **49**:44–8. doi:10.1026/0932-4089.49.1.44
35. Dettmers J, Clauß E. "Arbeitsgestaltungskompetenzen für flexible und selbstgestaltete Arbeitsbedingungen,". In: Janneck M, Hoppe A, editors. *Gestaltungskompetenzen für gesundes Arbeiten*. Berlin, Heidelberg: Springer Berlin Heidelberg (2018). p. 13–25.
36. Rigotti T, Schyns B, Mohr G. A Short Version of the Occupational Self-Efficacy Scale: Structural and Construct Validity Across Five Countries. *Journal of Career Assessment* (2008) **16**:238–55. doi:10.1177/1069072707305763
37. Abler B, Kessler H. "ERQ - Emotion Regulation Questionnaire - deutsche Fassung,". In: Leibniz-Zentrum für Psychologische Information und Dokumentation, editor. *Elektronisches Testarchiv*. Trier: ZPID (2011).
38. Gusy, B., & Lohmann, K. "Berliner-Anforderungs Ressourcen Inventar für Studierende,". In: B. Gusy & K. Lohmann, editor. *Instrumentenhandbuch zum Projekt, University Health Report*. Berlin: Freie Universität Berlin: Schriftenreihe des Instituts für Prävention und psychosoziale Gesundheitsforschung (Nr 01/P14) (2014). p. 31–40.

39. Fend H. *Der Umgang mit der Schule in der Adoleszenz [Coping with school during adolescence]*. Bern: Huber (1997).

40. Gusy B, Wörfel F, Lohmann K. Erschöpfung und Engagement im Studium. *Zeitschrift für Gesundheitspsychologie* (2016) **24**:41–53. doi:10.1026/0943-8149/a000153

41. Krause A, Baeriswyl S, Berset M, Deci N, Dettmers J, Dorsemagen C, et al. Selbstgefährdung als Indikator für Mängel bei der Gestaltung mobil-flexibler Arbeit: Zur Entwicklung eines Erhebungsinstruments. *Wirtschaftspsychologie* (2015).

42. Daniels K, Beesley N, Cheyne A, Wimalasiri V. Coping processes linking the demands-control-support model, affect and risky decisions at work. *Human Relations* (2008) **61**:845–74. doi:10.1177/0018726708093543
